# Molecular characterization of serotype and virulence genes of Pseudomonas aeruginosa isolated from patients admitted at two hospitals in Addis Ababa, Ethiopia

**DOI:** 10.1099/jmm.0.002034

**Published:** 2025-06-24

**Authors:** Matifan Dereje Olana, Daniel Asrat, Göte Swedberg

**Affiliations:** 1 Department of Medical Biochemistry and Microbiology, Biomedical Centre, Uppsala University, Uppsala, Sweden; 2 Department of Microbiology, Immunology and Parasitology, College of Health Sciences, Addis Ababa University, Addis Ababa, Ethiopia; 3 Department of Medical Laboratory Sciences, College of Medicine and Health Sciences, Ambo University, Ambo, Ethiopia

**Keywords:** Ethiopia, *Pseudomonas aeruginosa*, serotypes, virulence genes, whole-genome sequencing

## Abstract

**Introduction.** *Pseudomonas aeruginosa* contains a wide range of extracellular and cell-associated virulence factors that support its pathogenesis. The most variable portion of lipopolysaccharide, O-polysaccharide, confers serogrouping and is crucial for virulence.

**Gap Statement.** Despite their importance, *P. aeruginosa* serotypes and associated virulence factors are not well described at the level of strains obtained from Ethiopian clinical samples.

**Aim.** To characterize the serotypes and virulence factors of *P. aeruginosa* isolates from patients admitted to two hospitals in Addis Ababa, Ethiopia.

**Methodology.** Whole-genome sequencing was performed to characterize genes responsible for serotypes and virulence factors.

**Results.** Eight distinct serotypes were identified, with O6 (50%) and O11 (14.1%) being the most common and O9 (1.6%) being the least common. Serotype O6 was the most frequent serotype in all infections, and the percentage of O11 (38.5%) was high in burn wound isolates. The percentage of multidrug resistance was 56.6%. High levels of resistance to ciprofloxacin (51.8%) and ceftazidime (50.6%) and low levels of resistance to ceftazidime-avibactam (4.8%) were observed. Multidrug-resistant phenotypes were more common for the O11 (88.9%) and O5 (66.7%) serotypes. There were four (6.3%) *exoU+* strains and one (1.6%) *exoU+exoS*+ multidrug-resistant strain, all of which were O11 serotypes. The frequencies of *toxA*, *exoY*, *pilA* and *exoT* were 93.8%, 96.9%, 17.2% and 96.9 %, respectively.

**Conclusion.** This study showed the presence of highly virulent multidrug-resistant *P. aeruginosa* strains in Ethiopia, and continuous molecular surveillance is essential for monitoring the spread of these strains and creating efficient management strategies.

## Data Summary

Sequence data that support the findings of this study have been deposited in online repositories. The accession number(s) can be found in Table SB are available in the online Supplementary Material.

## Introduction

*Pseudomonas aeruginosa* is a gram-negative bacterium that can adapt to a wide range of environments and has a diverse metabolism. It is one of the main causes of hospital-acquired infections (HAIs), particularly in immunocompromised individuals [1].

The pathogenicity of *P. aeruginosa* is associated with the expression of multiple virulence factors [2]. Certain virulence factors facilitate the colonization of bacteria on the host surface, whereas others accelerate the invasion of several tissues [2,3]. Type IV pili play a vital role in bacterial attachment and initial colonization of mucosal cell surfaces [3]. During infection, *P. aeruginosa* can produce and release a variety of toxins, including four that are linked to the type III secretion system (T3SS), namely, *ExoS*, *ExoU*, *ExoT* and *ExoY* [4]. *ExoU* is the most toxic effector protein delivered by the T3SS and is associated with increasing disease severity in *P. aeruginosa* infections [5].

Pyoverdine and pyochelin are amongst the virulence factors involved in chronic infection, whereas exotoxin A and phospholipase C cause tissue necrosis and thermolabile haemolysis, respectively [6]. Proteases released by *P. aeruginosa*, such as elastase A, elastase B, protease IV, alkaline protease, MucD and *P. aeruginosa* aminopeptidase, have strong proteolytic enzyme activity and are responsible for protein degradation that damages host tissues [2].

Lipopolysaccharide (LPS) is a crucial surface structural element that protects the exterior layer of the bacterial membrane and interacts with host cells. The structure of LPS is composed of lipid A, which is covalently attached to a core oligosaccharide and capped by the O antigen [7]. Different bacterial species exhibit heterogeneous chemical compositions of the O antigens, which are extremely varied and play crucial roles in host‒pathogen interactions [8]. *P. aeruginosa* can be classified into 20 different O serotypes (O1 to O20) on the basis of the structure of their O antigens [9], with O6 and O11 being amongst the most prevalent [10,11]. Serotypes O12, O4 and O11 are frequently reported from multidrug-resistant (MDR) phenotypes [12].

The virulence factors of *P. aeruginosa* can be associated with increasing severity of infection and pathogenicity [2]. Understanding the specific serotypes and virulence factors of *P. aeruginosa* in a local context provides insight into its virulence mechanism and contributes to the global understanding of this pathogen. Despite their importance, strains identified from Ethiopian clinical samples have not been thoroughly characterized for serotypes and virulence through whole-genome sequencing (WGS). The purpose of this study was to characterize the serotypes and virulence potential of *P. aeruginosa* strains isolated from patients admitted to Tikur Anbessa Specialized Hospital (TASH) and Yekatit 12 Medical College Hospital (Y12HMC) in Addis Ababa, Ethiopia. To the best of our knowledge, this is the first study to characterize the serotypes and virulence potential of *P. aeruginosa* clinical strains in Ethiopia.

## Methodology

### *P. aeruginosa* isolation

Between August 2022 and August 2023, a cross-sectional study was conducted at TASH and Y12HMC. Culture, identification and quality control of conventional tests were conducted according to our previous study [13].

### Antibiotic susceptibility test

Antimicrobial susceptibility testing (AST) was assessed via Kirby–Bauer disc diffusion according to CLSI guidelines [14]. AST was tested against imipenem (IMP, 10 µg), meropenem (MEM, 10 µg), gentamicin (CN, 10 µg), netilmicin (NET, 30 µg), ciprofloxacin (CIP, 5 µg), levofloxacin (LEV, 5 µg), ceftazidime (CAZ, 30 µg), cefepime (FEP, 30 µg), ceftazidime-avibactam (CZA, 30/20 µg), piperacillin-tazobactam (PTZ, 100/10 µg) and aztreonam (ATM, 30 µg). The isolates were categorized as MDR or non-MDR strains on the basis of the criteria described by Magiorakos *et al*. [15].

### DNA extraction and WGS

DNA was extracted via a QIAGEN extraction kit according to the manufacturer’s instructions. The sequencing libraries were created via Nextera XT Illumina kits, and short-read sequencing was performed via a 150 bp insert size paired-end sequencing protocol on an Illumina HiSeq 2500 system (Illumina, San Diego, CA, USA) at Science for Life Laboratory, Solna, Sweden.

### Assembly and quality assessing

The quality of the paired-end short reads from Illumina sequencing was evaluated via fastq v0.12.1 [16]. Adapters, unreliable reads and low-quality sequences were trimmed via fastp v0.23.4 [17]. Assembly of the raw readings was performed via SPAdes version 3.15.0 [18]. Species identification via WGS was carried out via JSpeciesWS v3.2.7 [19]. Contaminated or mixed sequences and isolates with poor sequencing quality were removed from further analysis.

### Serotype and virulence gene detection

Virulence genes were identified from *de novo* assemblies via abricate v1.0.1 [20], with searches performed against the Virulence Factor Database [21]. Serotyping of the *P. aeruginosa* strains was performed via Past v1.0 software (CGE Server (dtu.dk) [22].

### Phylogenetic analysis

The phylogenetic tree was constructed by aligning a set of genomes against the reference genome [*P. aeruginosa PAO1* (SAMN02603714)] and *P. aeruginosa DK2* (SAMN02603895) via core genome SNP analysis with parsnp v2.0.6 [23]. The phylogenetic tree was visualized and annotated with metadata via Chiplot [24].

## Results

### Prevalence of *P. aeruginosa* isolates

Amongst the 422 clinical samples processed during the study period, 83 (19.6 %) *P*. *aeruginosa* isolates were identified, with 36 from Y12HMC and 47 from TASH. The prevalence rates of isolates from blood, urine, burn and surgical site wounds were 19 (22.9%), 27 (32.5 %), 14 (16.9%) and 23 (27.7 %), respectively. Following the sequencing of all 83 isolates, isolates with poor read coverage, high error rates or contamination were excluded, and 64 isolates were subjected to molecular characterization for serotype and virulence detection to ensure high-quality and informative sequencing results.

## AST profile

*P. aeruginosa* presented the greatest degree of resistance to CIP (51.8%), CAZ (50.6%) and FEP (48.2%). The rate of CZA resistance was low (4.8%) (Table 1).

**Table 1.** Susceptibility rates of *P. aeruginosa* to different antibiotics

AST profile (%)	MEM	IMP	CN	NET	CIP	LEV	CAZ	FEP	PTZ	CZA	ATM
R	22.9	16.9	43.4	26.5	51.8	37.3	50.6	48.2	22.9	4.8	42.2
I	12	3.6	0	2.4	12	3.6	15.7	16.9	1.2	0	0
S	65.1	79.5	56.6	71.1	36.1	59	34.9	34.9	75.9	95.2	57.8

ATM, aztreonam; CAZ, ceftazidime; CIP, ciprofloxacin; CN, gentamicin; CZA, ceftazidime-avibactam; FEP, cefepime; I, intermediate; IMP, imipenem; LEV, levofloxacin; MEM, meropenem; NET, netilmicin; PTZ, piperacillin-tazobactam; R, resistant; S, susceptible.

### Prevalence of *P. aeruginosa* serotypes

**Fig. 1.**
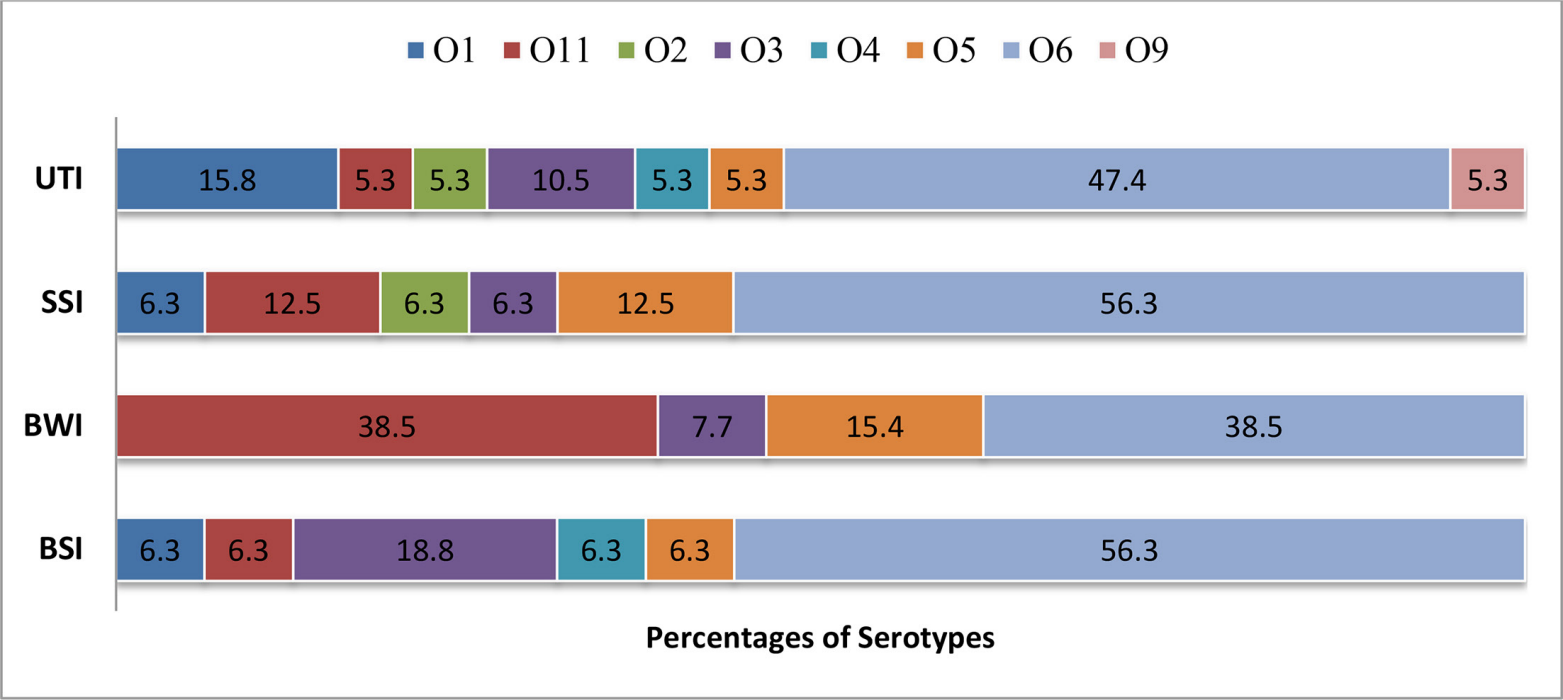
Percentages of *P. aeruginosa* serotypes amongst different types of infections. BSI, bloodstream infection; BWI, burn wound infection; SSI, surgical site infection; UTI, urinary tract infection.

Eight different serotypes were identified amongst the 64 isolates. Overall, the most prevalent serotypes were O6 (50%), O11 (14.1%), O3 (10.9%), O5 (9.4%), O1 (7.8%), O2 (3.1%) and O4 (3.1%), with O9 (1.6%) being the least common. In terms of infections, serotype O6 was the most frequently found serotype, accounting for 47.4%, 56.3%, 38.5% and 56.3% of isolates from blood, surgical wounds, burn wounds and urine, respectively (Fig. 1). Serotype O1 was the second most prevalent serotype amongst the urine isolates (15.8%), serotype O3 was the second most prevalent serotype amongst the blood isolates and O11 was more common amongst the surgical wound (12.5%) and burn wound (38.5%) isolates (Fig. 1).

### Serotypes and MDR phenotypes

**Fig. 2.**
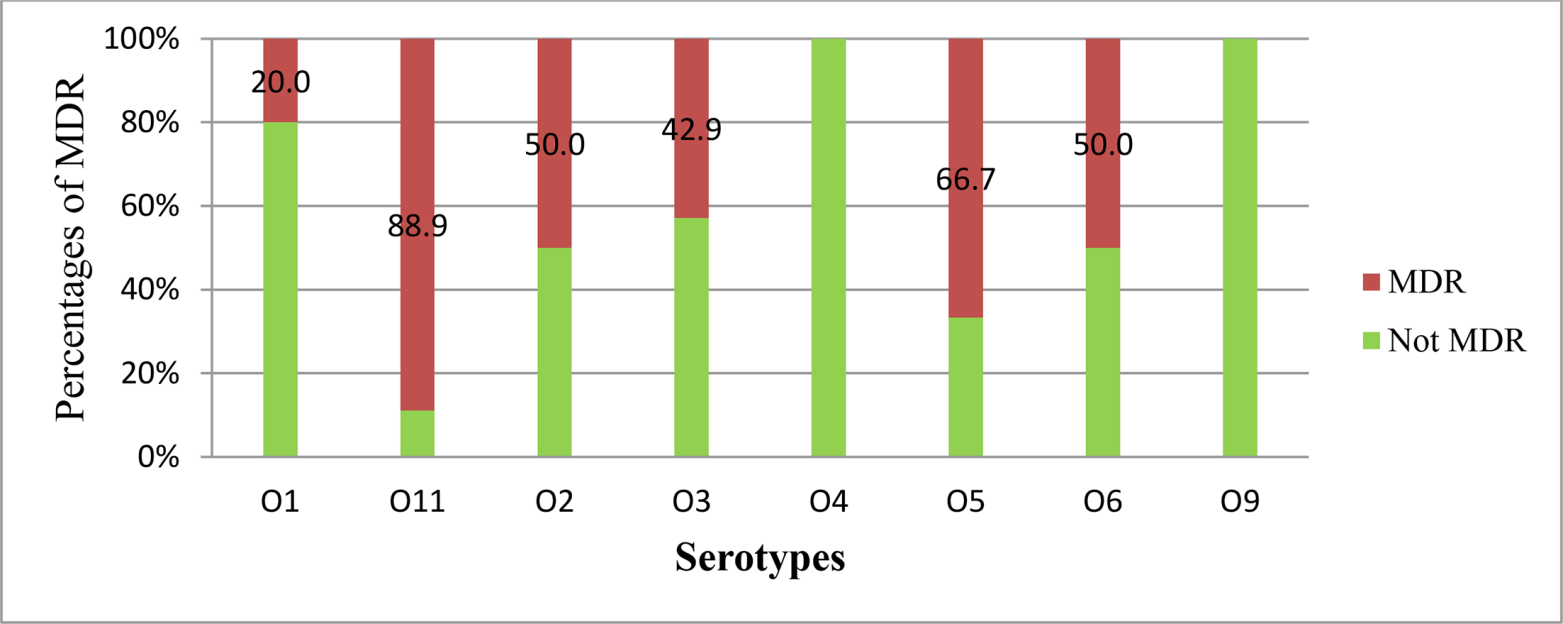
Percentages of MDR phenotypes amongst *P. aeruginosa* serotypes.

Fig. 2 shows the distribution of MDR and non-MDR percentages amongst *P. aeruginosa* serotypes. Each bar with two segments indicates MDR and non-MDR percentages, which represent specific serotypes. Certain serotypes had high percentages of MDR, whereas others did not, indicating varying resistance levels across serotypes. Serotype O11 had the highest percentage of MDR strains (88.9%), followed by serotype O5 (66.7%). Serotypes O6 and O2 had equal distributions (50%) of MDR and non-MDR percentages, whereas serotypes O4 and O9 had 100% non-MDR isolates (**Fig. 2**).

### Distribution of MDR and *exoU* toxin genes across serotypes

**Fig. 3.**
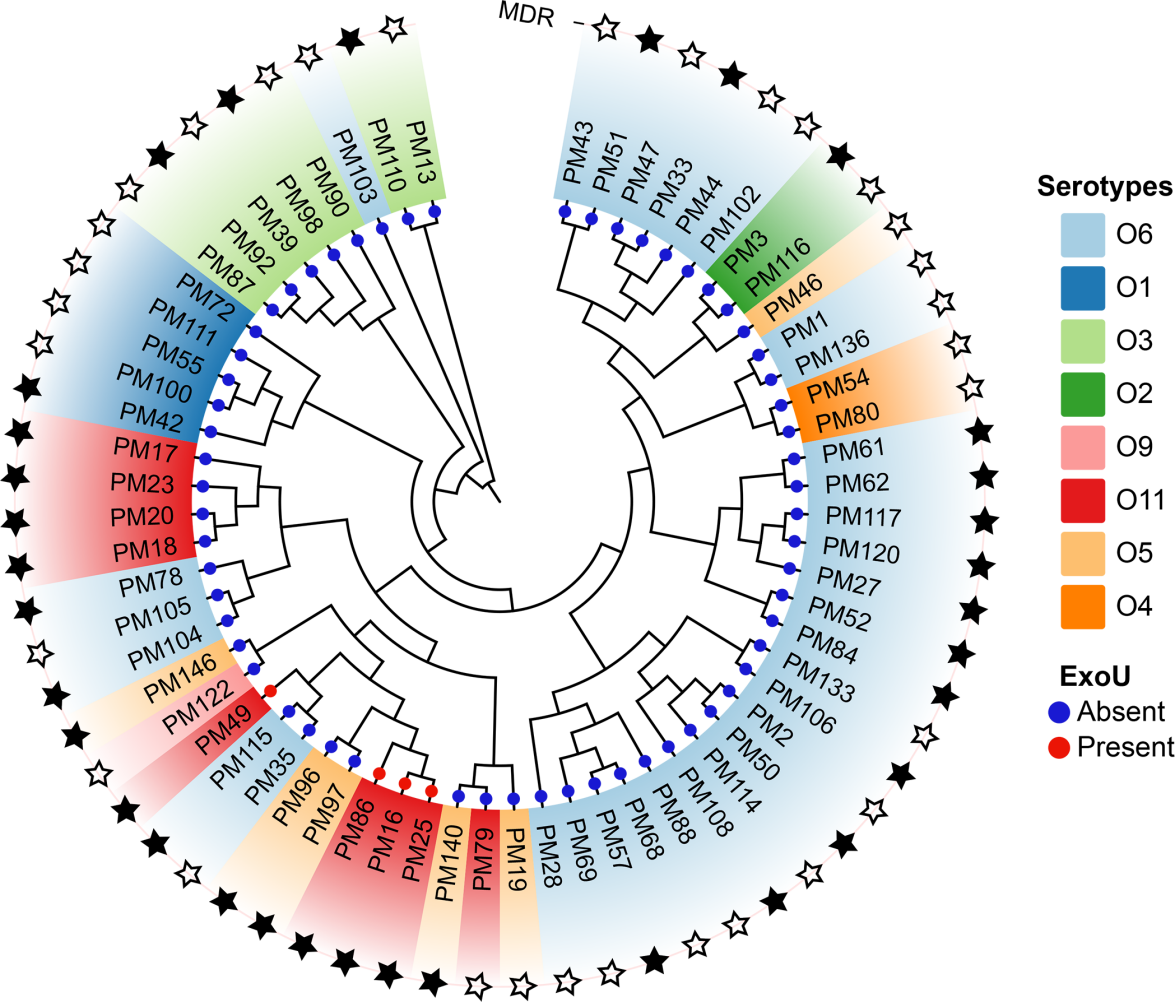
Phylogenetic clustering and distribution of MDR and *exoU* toxin genes in *P. aeruginosa* serotypes. The black stars represent MDR strains, the white stars represent non-MDR strains, the red circles represent *exoU*-positive strains and the blue circles represent *exoU*-negative strains with different bands of similar colour, indicating strains with the same serotypes.

Phylogenetic analysis revealed that most isolates of the same serotype clustered under the same clade and exhibited similar MDR profiles, indicating genetic relatedness. However, some serotypes in the same clade exhibited different MDR profiles, indicating possible variation in resistance acquisition amongst serotypes. Our study revealed that all isolates with the presence of *exoU* toxin were MDR isolates, and all belonged to serotype O11 (Fig. 3).

### Prevalence of virulence genes

Amongst the 241 virulence genes identified, 83.4% were present in nearly every isolate. Examples of these frequent virulence genes were flagella-related genes (*fliF* and *fliQ)*, type 2 secretion system components (*xcpZ* and *xcpR*), T3SS components (*pscN* and *pscP)*, T6SS-related genes (*clpV1* and *vgrG1a*) and secreted factors (*pvdG* and *phzm*). The most common toxins identified were *exoY* (96.9%), *exoT* (96.8%), *exoS* (95.3%) and *toxA* (93.8%). A comprehensive list of the virulence genes identified is provided in Table SA, available in the online Supplementary Material.

### Distribution of virulence genes across serotypes

**Fig. 4.**
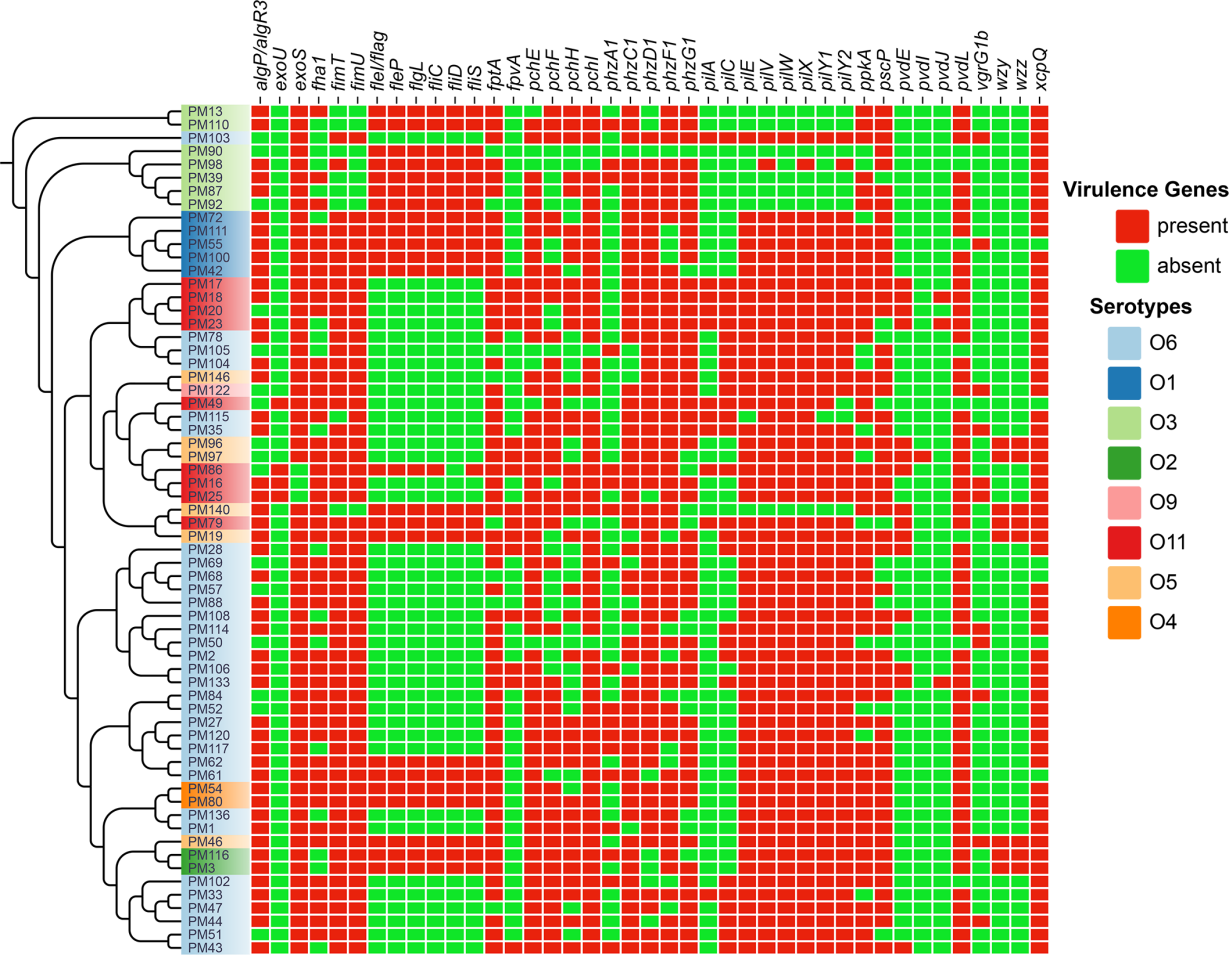
Phylogenetic clustering and distribution of variable virulence genes in *P. aeruginosa* strains. Isolates of the same serotypes are highlighted with the same colour. The presence and absence of virulence genes are shown in red and green, respectively.

The majority of genes associated with pilus and flagella were found in every isolate, whilst the prevalence of *fleP*, *fliS*, *fleI/flag*, *fliC* and *flgL* was 35.9% (Fig. 4). These genes were present in all O2, O3 and O4 serotypes, but only in 6.3% of O6 and 22.2% of O11 serotypes. The rare virulence factors include *pvdI*, which was found in one isolate of the O5 serotype, and *pvdJ*, which was found in three isolates, two of which were O11 and one of which was O6. The *exoU* toxin gene was found in four isolates. Of these, one showed co-presence of *exoU* and *exoS*, and all belonged to serotype O11. All other isolates carried the *exoS* gene. Only eight (12.5%) isolates contained *wzz* and *wzy* genes, which encode enzymes involved in the biosynthesis and modification of LPSs. Amongst these serotypes, five (62.5%) were O5, two (25%) were O2 and one (12.5%) was O11. Only 11 (17.8%) isolates contained *pilA* genes, with 63.6% in O11 and 36.4% in O6. Other virulence genes present in a few isolates were *vgrG1b* in 11 (17.8%) and *phzA1* in 18 (28%), and these were more frequently found in O6 serotypes, with 54.5% and 61.1%, respectively. A key factor in biofilm formation, *algP/algR3*, was detected in 48 isolates (75%) and was present in almost all of the identified serotypes, indicating its broad distribution amongst various serotypes (Fig. 4).

## Discussion

*P. aeruginosa* is the major cause of HAIs and is often associated with increasing MDR [13]. It is a dynamic pathogen with extensive genetic variability and virulence factors associated with its pathogenicity [25]. Given that antibiotic resistance in *P. aeruginosa* is complicating treatment options, a comprehensive understanding of serotypes and their virulence potential is crucial for effective therapeutic strategies and control measures. To the best of our knowledge, this is the first study in Ethiopia to characterize the serotypes and virulence potential of *P. aeruginosa* isolates from patients admitted to two hospitals in Addis Ababa, Ethiopia.

In the present study, O6 (50%) and O11 (14.1%) were the most common serotypes. Similarly, O6 and O11 were amongst the most common in China (29.13 % vs. 23.3 %) [26], Spain (17.8 % vs. 13.3%) [12] and France (26% vs. 23%) [10]. Compared with our study, where 75% of the isolates were O6, O11 or O3, the WGS of more than 1,000 *P*. *aeruginosa* strains revealed that 70% of the isolates in the prior study were O3, O6, O11 or O12 [22]. Geographical differences, serotyping techniques, the number of isolates tested and genetic adaptation can all contribute to variations in the number and type of serotypes. Generally, the predominant serotypes of *P. aeruginosa* isolates in our study were not significantly different from the predominant serotypes reported in other countries [12,26].

Serotypes O3 (18.8%) and O11 (38.5%) were more prevalent in bloodstream infections and burn wound infections, respectively, whereas O6 was the most common of all infections. Previous serotyping data indicate that serotypes O6 (29%) and O11 (23%) are the most common in *P. aeruginosa* nosocomial pneumonia [10] and other types of infections [26]. Serotype O9 was a rare finding identified in only one isolate, consistent with previous studies [22]. Although being a rare serotype, previous studies reported that serotype O9 can elicit a strong immune response and may serve as a promising candidate for vaccine development; however, its potential is limited by the acid-labile nature of its polysaccharides [27]. Some *P. aeruginosa* serotypes may be more adaptable to specific tissues because of differences in their O-specific antigen, which could increase their persistence in particular infections [28].

The percentage of MDR in *P. aeruginosa* was 56.6%. This finding is lower than those of previous studies conducted in Ethiopia (80.5%) [29], Brazil (76.2%) [30] and Egypt (70%) [31] and higher than those reported in Ethiopia (45.9%) [32], Kenya (31%) [33], Iraq (50%) [34] and China (22.3%) [35]. A high resistance rate for CIP (51.8%), followed by CAZ (50.6%) and a low resistance rate to CZA (4.8%), was identified. Comparable findings for CIP and CZA were reported in Ethiopia [29] and China [36], whereas lower resistance rates to CIP (15.4%) and CAZ (37.7%) were reported in China [37] and Iran [38]. The resistance levels to MEM and IMP were 22.9% and 16.9%, respectively. Comparable findings of MEM resistance were reported in Ethiopia (28.6%) [29], and higher resistance levels to MEM were reported in India (80%) [39]. Similarly, a relatively high resistance level to IMP was reported in Iran (70%) [40]. The discrepancies in resistance between studies may be due to socioeconomic conditions, local healthcare practices and variations in diagnostic techniques [37,39].

Serotype O11 was the most common serotype of MDR isolates, which is comparable to the findings of a previous study [41]. In our study, O4 was found only amongst non-MDR isolates, and O12 was not identified; however, contrasting findings were reported in Spain, where MDR phenotypes were more frequent for O4 (57.3%) [12]. Previous studies have shown that serotype O12 has become the most common serotype in clinical settings and shows high levels of resistance to different antibiotic classes [12,41]. Despite the fact that serotypes O11, O12 and O4 accounted for the majority of reported MDR phenotypes, variations in MDR patterns amongst studies may be due to differences in strain distribution, geographic location and the dynamic nature of *P. aeruginosa*.

*P. aeruginosa* has a number of virulence factors that could be involved in its pathogenicity [25]. Using data from hundreds of *Pseudomonas* genomes, the Pseudomonas Genome Database (http://www.pseudomonas.com) has documented over 320 virulence genes [42]. In the present study, 241 virulence genes were identified. Although our results are consistent with those of previous studies [33], possibly because both studies used the same methodology (WGS-based), virulence genes still vary because of the methodological variation and genetic diversity of *P. aeruginosa* strains. This diversity and variation can be associated with diversity in colonization, tissue damage and infection severity, influencing its pathogenicity [5]. In the present study, the biofilm-related gene *algP/algR3* was found in 75% of the isolates, and the flagella-related genes *fleP*, *fliS*, *fleI* and *flgL* were identified in 35.9% of the isolates, which is comparable with studies conducted in Kenya [33] and Saudi Arabia [43]. Analysis of the current dataset revealed that the frequency of *pilA* was 17.2%, which is relatively comparable with the finding reported in a study conducted in Iran (24.7%) [44]. The highest frequencies were for *exoY* (96.9%) and *exoT* (96.8%), followed by *exoS* (95.3%) and *toxA* (93.8%). Comparable results were reported regarding the frequency of *toxA* (97.8%) and *exoY* (93.1%) in Iran [44], whereas a higher prevalence rate for *exoT* (83%) was reported in another study [45]. In general, the frequency of virulence genes identified here was not significantly different from the frequency of virulence genes reported in other studies [33]. However, minor variations across the studies may be attributed to variability in the methodological techniques employed, such as PCR-based or genome sequencing approaches [43,46].

The *exoU* toxin was found in 6.3% of the *P. aeruginosa* clinical strains. A meta-analysis revealed that 32% of clinical *P. aeruginosa* isolates carried *exoU* toxin genes [45], whereas a study carried out in Spain reported a figure of 31.1% [4]. Furthermore, *exoU* toxin was reported in *P. aeruginosa* strains from food products (2.5%) in Côte d’Ivoire [46] and drinking water (7.6%) in China [47]. All the *exoU*-positive strains were serotyped as O11, which is comparable with the results of a previous study [5]. All O11 strains harbouring *exoU* were MDR isolates. Similarly, a relatively high *exoU* frequency in MDR strains was reported in a previous study [48]. The co-occurrence of *exoU* with MDR traits in serotype O11 strains suggests the convergence of virulence and resistance, which poses serious therapeutic challenges [49]. Such strains not only have an enhanced capacity to cause severe infections but also lead to limited treatment options and poor clinical outcomes [50]. *ExoU* is the most intensely cytotoxic of the four effector proteins and has phospholipase activity; it is capable of rupturing host cell membranes and compromising the innate immune response to infection [51]. *ExoU* induces strong inflammatory responses [52] and has been studied as a potential therapeutic target [53]. When the *ExoU* gene is expressed in *P. aeruginosa* infections, the prognosis is worse, and it is associated with increased virulence, tissue injury [5], disease severity and mortality rate [54]. Although studies have reported greater *exoU* presence in O11, further molecular investigation is needed to clarify the mechanism behind the connections between *exoU* and O-antigen types. Furthermore, despite the fact that serotype O6 is so prevalent, infections caused by serotype O6 often result in lower severity and better clinical outcomes as compared to serotype O11 [10,26]. Notably, serotype O6 strains are often linked to the presence of the *exoS* gene and commonly associated with a lack of *exoU* toxin, and that may contribute to the variability in clinical outcomes [10].

It is uncommon for one bacterial strain to possess both *ExoU* and *ExoS* [55]. This is hypothesized to be due to the deletion of *exoS* in strains that acquire the *exoU/spcU* locus resulting from a targeted deletion event caused by a product of a gene linked to the *exoU/spcU* region at the time of acquisition, even if the exact mechanism is not clear [56]. Despite the rarity of finding both toxins in a single strain, they have been documented, which can be attributed to the genetic complexity of *P. aeruginosa*. In our study, one strain carried both *ExoU* and *ExoS*; similarly, single hypervirulent strains carrying both toxins were reported in previous studies [26,50]. According to previous findings, the coexpression of *exoU* and *exoS* toxins enhances the cytotoxicity and pathogenicity of *P. aeruginosa* strains, and it is likely that horizontal gene transfer of the pathogenicity island PAPI-2 promotes *exoU* acquisition by *exoS+ P. aeruginosa* [50]. Our findings of a *P. aeruginosa* strain that possesses both *exoU* and *exoS* show the existence of a highly virulent strain that might cause serious infections.

## Conclusion

In the present study, we identified eight distinct serotypes of *P. aeruginosa*, as well as a number of virulence genes linked to pathogenicity. Serotype O6 was the most frequently found serotype. We found highly virulent strains that harboured *exoU* toxin and both *exoU* and *exoS* in the O11 serotype. This finding revealed the presence of a highly virulent strain in Ethiopia and emphasized the importance of ongoing molecular surveillance to track the further spread of such strains.

## Supplementary material

10.1099/jmm.0.002034 Uncited Supplementary Material 1.

## Abbreviations

AST: antimicrobial susceptibility testing
ATM: aztreonam
BSI: blood steam infection
CAZ: ceftazidime
CIP: ciprofloxacin
CN: gentamicin
CZA: ceftazidime-avibactam
FEP: cefepime
HAI: hospital-acquired infections
IMI: imipenem
LEV: levofloxacin
LPS: lipopolysaccharide
MDR: multidrug-resistant
MEM: meropenem
NET: netilmicin
PTZ: piperacillin-tazobactam
SSI: surgical site infection
TASH: Tikur Anbessa Specialized Hospital
T3SS: type III secretion system
WGS: whole-genome sequencing
Y12HMC: Yekatit 12 Medical College Hospital
