# Supplementary material for: Molecular characterization of serotype and virulence genes of Pseudomonas aeruginosa isolated from patients admitted at two hospitals in Addis Ababa, Ethiopia
Source: J Med Microbiol. 2025 Jun 24;74(6):002034. doi: 10.1099/jmm.0.002034 (PMC12451762; doi:10.1099/jmm.0.002034)
Supplement: Uncited Supplementary Material 1. [file jmm-74-02034-s001.pdf]

Supplementary table A: List of Virulence Gene Identified among *Pseudomonas aeruginosa* isolated from patients admitted at TASH and Y12HMC hospitals in Addis Ababa, Ethiopia.

No	Virulence Gene	Product
1.	*alg44*	Alginate
2.	*alg8*	Alginate
3.	*algA*	Alginate
4.	*algB*	Alginate
5.	*algC*	Alginate
6.	*algD*	Alginate
7.	*algE*	Alginate
8.	*algF*	Alginate
9.	*algG*	Alginate
10.	*algI*	Alginate
11.	*algJ*	Alginate
12.	*algK*	Alginate
13.	*algL*	Alginate
14.	*algP/algR3*	Alginate
15.	*algQ*	Alginate
16.	*algR*	Alginate
17.	*algU*	Alginate
18.	*algW*	Alginate
19.	*algX*	Alginate
20.	*algZ*	Alginate
21.	*aprA*	Alkaline Protease

22.	*chpA*	Type IV pili
23.	*chpB*	Type IV pili
24.	*chpC*	Type IV pili
25.	*chpD*	Type IV pili
26.	*chpE*	Type IV pili
27.	*clpV1*	Type IV pili
28.	*dotU1*	Type III secretion system
29.	*exoS*	TTSS secreted effectors
30.	*exoT*	TTSS secreted effectors
31.	*exoU*	TTSS secreted effectors
32.	*exoY*	TTSS secreted effectors
33.	*exsA*	Type III secretion system
34.	*exsB*	Type III secretion system
35.	*exsC*	Type III secretion system
36.	*exsD*	Type III secretion system
37.	*exsE*	Type III secretion system
38.	*fha1*	Filamentous hemagglutinin protein
39.	*fimT*	Type IV pili
40.	*fimU*	Type IV pili
41.	*fimV*	Type IV pili
42.	*fleI/flag*	Flagella
43.	*fleN*	Flagella
44.	*fleP*	Flagella

45.	*fleQ*	Flagella
46.	*fleR*	Flagella
47.	*fleS*	Flagella
48.	*flgA*	Flagella
49.	*flgB*	Flagella
50.	*flgC*	Flagella
51.	*flgD*	Flagella
52.	*flgE*	Flagella
53.	*flgF*	Flagella
54.	*flgG*	Flagella
55.	*flgH*	Flagella
56.	*flgI*	Flagella
57.	*flgJ*	Flagella
58.	*flgK*	Flagella
59.	*flgL*	Flagella
60.	*flgM*	Flagella
61.	*flgN*	Flagella
62.	*flhA*	Flagella
63.	*flhB*	Flagella
64.	*flhF*	Flagella
65.	*fliA*	Flagella
66.	*fliC*	Flagella
67.	*fliD*	Flagella

68.	*fliE*	Flagella
69.	*fliF*	Flagella
70.	*fliG*	Flagella
71.	*fliH*	Flagella
72.	*fliI*	Flagella
73.	*fliJ*	Flagella
74.	*fliK*	Flagella
75.	*fliL*	Flagella
76.	*fliM*	Flagella
77.	*fliN*	Flagella
78.	*fliO*	Flagella
79.	*fliP*	Flagella
80.	*fliQ*	Flagella
81.	*fliR*	Flagella
82.	*fliS*	Flagella
83.	*fptA*	Flagella
84.	*fpvA*	Flagella
85.	*hcp1*	Type VI secretion system
86.	*hsiA1*	Type VI secretion system
87.	*hsiB1/vipA*	Type VI secretion system
88.	*hsiC1/vipB*	Type VI secretion system
89.	*hsiE1*	Type VI secretion system
90.	*hsiF1*	Type VI secretion system

91.	*hsiG1*	Type VI secretion system
92.	*hsiH1*	Type VI secretion system
93.	*hsiJ1*	Type VI secretion system
94.	*icmF1/tssM1*	Type VI secretion system
95.	*lasA*	Serine protease
96.	*lasB*	Serine protease
97.	*lasI*	Serine protease
98.	*lip1*	Lipase enzyme
99.	*mbtH-like*	Biosynthesis of secondary metabolites
100.	*motA*	Flagella
101.	*motB*	Flagella
102.	*motC*	Flagella
103.	*motD*	Flagella
104.	*motY*	Flagella
105.	*mucA*	Regulator alginate biosynthesis
106.	*mucB*	Regulator alginate biosynthesis
107.	*mucC*	Regulator alginate biosynthesis
108.	*mucD*	Regulator alginate biosynthesis
109.	*mucE*	Regulator alginate biosynthesis
110.	*mucP*	Regulator alginate biosynthesis
111.	*pchA*	Pyochelin
112.	*pchB*	Pyochelin
113.	*pchC*	Pyochelin

114.	*pchD*	Pyochelin
115.	*pchE*	Pyochelin
116.	*pchF*	Pyochelin
117.	*pchG*	Pyochelin
118.	*pchH*	Pyochelin
119.	*pchI*	Pyochelin
120.	*pchR*	Pyochelin
121.	*pcr1*	Type III secretion system
122.	*pcr2*	Type III secretion system
123.	*pcr3*	Type III secretion system
124.	*pcr4*	Type III secretion system
125.	*pcrD*	Type III secretion system
126.	*pcrG*	Type III secretion system
127.	*pcrH*	Type III secretion system
128.	*pcrR*	Type III secretion system
129.	*pcrV*	Type III secretion system
130.	*phzA1*	Pyocyanin
131.	*phzB1*	Pyocyanin
132.	*phzC1*	Pyocyanin
133.	*phzD1*	Pyocyanin
134.	*phzE1*	Pyocyanin
135.	*phzF1*	Pyocyanin
136.	*phzG1*	Pyocyanin

137.	*phzH*	Pyocyanin
138.	*phzM*	Pyocyanin
139.	*phzS*	Pyocyanin
140.	*pilA*	Type IV pili
141.	*pilB*	Type IV pili
142.	*pilC*	Type IV pili
143.	*pilE*	Type IV pili
144.	*pilF*	Type IV pili
145.	*pilG*	Type IV pili
146.	*pilH*	Type IV pili
147.	*pilI*	Type IV pili
148.	*pilJ*	Type IV pili
149.	*pilK*	Type IV pili
150.	*pilM*	Type IV pili
151.	*pilN*	Type IV pili
152.	*pilO*	Type IV pili
153.	*pilP*	Type IV pili
154.	*pilQ*	Type IV pili
155.	*pilR*	Type IV pili
156.	*pilS*	Type IV pili
157.	*pilT*	Type IV pili
158.	*pilU*	Type IV pili
159.	*pilV*	Type IV pili

160.	*pilW*	Type IV pili
161.	*pilX*	Type IV pili
162.	*pilY1*	Type IV pili
163.	*pilY2*	Type IV pili
164.	*plcH*	Phospholipase C
165.	*popB*	Type III secretion system
166.	*popD*	Type III secretion system
167.	*popN*	Type III secretion system
168.	*ppkA*	Type III secretion system
169.	*pppA*	Type III secretion system
170.	*pscB*	Type III secretion system
171.	*pscC*	Type III secretion system
172.	*pscD*	Type III secretion system
173.	*pscE*	Type III secretion system
174.	*pscF*	Type III secretion system
175.	*pscG*	Type III secretion system
176.	*pscH*	Type III secretion system
177.	*pscI*	Type III secretion system
178.	*pscJ*	Type III secretion system
179.	*pscK*	Type III secretion system
180.	*pscL*	Type III secretion system
181.	*pscN*	Type III secretion system
182.	*pscO*	Type III secretion system

183.	*pscP*	Type III secretion system
184.	*pscQ*	Type III secretion system
185.	*pscR*	Type III secretion system
186.	*pscS*	Type III secretion system
187.	*pscT*	Type III secretion system
188.	*pscU*	Type III secretion system
189.	*ptxR*	Regulator of pseudotoxin
190.	*pvcA*	Pyoverdine
191.	*pvcB*	Pyoverdine
192.	*pvcC*	Pyoverdine
193.	*pvcD*	Pyoverdine
194.	*pvdA*	Pyoverdine
195.	*pvdE*	Pyoverdine
196.	*pvdF*	Pyoverdine
197.	*pvdG*	Pyoverdine
198.	*pvdH*	Pyoverdine
199.	*pvdI*	Pyoverdine
200.	*pvdJ*	Pyoverdine
201.	*pvdL*	Pyoverdine
202.	*pvdM*	Pyoverdine
203.	*pvdN*	Pyoverdine
204.	*pvdO*	Pyoverdine
205.	*pvdP*	Pyoverdine

206.	*pvdQ*	Pyoverdine
207.	*pvdS*	Pyoverdine
208.	*rhlA*	Rhamnolipid
209.	*rhlB*	Rhamnolipid
210.	*rhlC*	Rhamnolipid
211.	*rhlI*	Rhamnolipid
212.	*tagF/pppB*	Glycosyltransferase enzyme
213.	*tagQ*	Glycosyltransferase enzyme
214.	*tagR*	Glycosyltransferase enzyme
215.	*tagS*	Glycosyltransferase enzyme
216.	*tagT*	Glycosyltransferase enzyme
217.	*toxA*	Exotoxin A
218.	*tse1*	Type VI secretion system
219.	*tse2*	Type VI secretion system
220.	*tse3*	Type VI secretion system
221.	*vgrG1a*	Type VI secretion system
222.	*vgrG1b*	Type VI secretion system
223.	*waaA*	Glycosyltransferase enzyme
224.	*waaC*	Glycosyltransferase enzyme
225.	*waaF*	Glycosyltransferase enzyme
226.	*waaG*	Glycosyltransferase enzyme
227.	*waaP*	Glycosyltransferase enzyme
228.	*wzy*	Polysaccharide polymerase

229.	*wzz*	Polysaccharide polymerase
230.	*xcpA/pilD*	Type IV pili
231.	*xcpP*	Type IV pili
232.	*xcpQ*	Type IV pili
233.	*xcpR*	Type IV pili
234.	*xcpS*	Type IV pili
235.	*xcpT*	Type IV pili
236.	*xcpU*	Type IV pili
237.	*xcpV*	Type IV pili
238.	*xcpW*	Type IV pili
239.	*xcpX*	Type IV pili
240.	*xcpY*	Type IV pili
241.	*xcpZ*	Type IV pili

Supplementary table B: Online respiratory accession number of *Pseudomonas aeruginosa* isolated from patients admitted at TASH and Y12HMC hospitals in Addis Ababa, Ethiopia

SN	Accession	BioProject	BioSample	SRA.filename
1.	SRR31777892	SAMN45903079	SUB14942133	PM136
2.	SRR31777629	SAMN45903063	SUB14941991	PM87
3.	SRR31777628	SAMN45903064	SUB14941991	PM88
4.	SRR31777637	SAMN45903073	SUB14941991	PM108
5.	SRR31777634	SAMN45903076	SUB14941991	PM115
6.	SRR31777636	SAMN45903074	SUB14941991	PM111
7.	SRR31777630	SAMN45903062	SUB14941991	PM80
8.	SRR31777641	SAMN45903069	SUB14941991	PM102
9.	SRR31777631	SAMN45903061	SUB14941991	PM79
10.	SRR31777633	SAMN45903077	SUB14941991	PM120
11.	SRR31777642	SAMN45903060	SUB14941991	PM78
12.	SRR31777626	SAMN45903066	SUB14941991	PM97
13.	SRR31777632	SAMN45903078	SUB14941991	PM133
14.	SRR31777624	SAMN45903068	SUB14941991	PM100
15.	SRR31777627	SAMN45903065	SUB14941991	PM92
16.	SRR31777639	SAMN45903071	SUB14941991	PM104
17.	SRR31777635	SAMN45903075	SUB14941991	PM114
18.	SRR31777640	SAMN45903070	SUB14941991	PM103
19.	SRR31777643	SAMN45903059	SUB14941991	PM72
20.	SRR31777625	SAMN45903067	SUB14941991	PM98
21.	SRR31777638	SAMN45903072	SUB14941991	PM106
22.	SRR31774662	SAMN45903040	SUB14941414	PM2
23.	SRR31774663	SAMN45903039	SUB14941414	PM1
24.	SRR31774648	SAMN45903044	SUB14941414	PM25
25.	SRR31774651	SAMN45903041	SUB14941414	PM13
26.	SRR31774655	SAMN45903055	SUB14941414	PM55
27.	SRR31774653	SAMN45903057	SUB14941414	PM62
28.	SRR31774652	SAMN45903058	SUB14941414	PM68
29.	SRR31774660	SAMN45903050	SUB14941414	PM43
30.	SRR31774654	SAMN45903056	SUB14941414	PM57
31.	SRR31774657	SAMN45903053	SUB14941414	PM47
32.	SRR31774647	SAMN45903045	SUB14941414	PM27
33.	SRR31774649	SAMN45903043	SUB14941414	PM19
34.	SRR31774661	SAMN45903049	SUB14941414	PM39
35.	SRR31774656	SAMN45903054	SUB14941414	PM54
36.	SRR31774646	SAMN45903046	SUB14941414	PM28
37.	SRR31774658	SAMN45903052	SUB14941414	PM46
38.	SRR31774644	SAMN45903048	SUB14941414	PM35
39.	SRR31774645	SAMN45903047	SUB14941414	PM33

40.	SRR31774650	SAMN45903042	SUB14941414	PM17
41.	SRR31774659	SAMN45903051	SUB14941414	PM44
42.	SRR31751153	SAMN45875113	SUB14937069	PM116
43.	SRR31751145	SAMN45875105	SUB14937069	PM86
44.	SRR31751155	SAMN45875103	SUB14937069	PM84
45.	SRR31751147	SAMN45875119	SUB14937069	PM146
46.	SRR31751141	SAMN45875109	SUB14937069	PM96
47.	SRR31751148	SAMN45875118	SUB14937069	PM140
48.	SRR31751150	SAMN45875116	SUB14937069	PM122
49.	SRR31751152	SAMN45875114	SUB14937069	PM117
50.	SRR31751138	SAMN45875112	SUB14937069	PM110
51.	SRR31751144	SAMN45875106	SUB14937069	PM90
52.	SRR31751140	SAMN45875110	SUB14937069	PM105
53.	SRR31749804	SAMN45875095	SUB14932238	PM42
54.	SRR31749806	SAMN45875093	SUB14932238	PM23
55.	SRR31749802	SAMN45875097	SUB14932238	PM49
56.	SRR31749808	SAMN45875083	SUB14932238	PM3
57.	SRR31749789	SAMN45875092	SUB14932238	PM20
58.	SRR31749790	SAMN45875091	SUB14932238	PM18
59.	SRR31749791	SAMN45875090	SUB14932238	PM16
60.	SRR31749801	SAMN45875098	SUB14932238	PM50
61.	SRR31749797	SAMN45875102	SUB14932238	PM69
62.	SRR31749799	SAMN45875100	SUB14932238	PM52
63.	SRR31749798	SAMN45875101	SUB14932238	PM61
64.	SRR31749800	SAMN45875099	SUB14932238	PM51
